# Low cancer suspicion following experience of a cancer ‘warning sign’

**DOI:** 10.1016/j.ejca.2015.07.014

**Published:** 2015-11

**Authors:** Katriina L. Whitaker, Kelly Winstanley, Una Macleod, Suzanne E. Scott, Jane Wardle

**Affiliations:** a School of Health Sciences, University of Surrey, Guildford, Surrey GU2 7XH, UK; b Health Behaviour Research Centre, Department of Epidemiology and Public Health, University College London, London WC1E 6BT, UK; c Centre for Health and Population Sciences, Hull York Medical School, Hull HU6 7RX, UK; d Unit of Social and Behavioural Sciences, King’s College London Dental Institute, London SE5 9RW, UK

**Keywords:** Socioeconomic factors, Cancer, Signs and symptoms, Delayed diagnosis

## Abstract

**Aim:**

Lower socioeconomic status (SES) is associated with a higher risk of late-stage cancer diagnosis. A number of explanations have been advanced for this, but one which has attracted recent attention is lower patient knowledge of cancer warning signs, leading to delay in help-seeking. However, although there is psychometric evidence of SES differences in knowledge of cancer symptoms, no studies have examined differences in ‘cancer suspicion’ among people who are actually experiencing a classic warning sign.

**Methods:**

A ‘health survey’ was mailed to 9771 adults (⩾50 years, no cancer diagnosis) with a symptom list including 10 cancer ‘warning signs’. Respondents were asked if they had experienced any of the symptoms in the past 3 months, and if so, were asked ‘what do you think caused it?’ Any mention of cancer was scored as ‘cancer suspicion’. SES was indexed by education.

**Results:**

Nearly half the respondents (1732/3756) had experienced a ‘warning sign’, but only 63/1732 (3.6%) mentioned cancer as a possible cause. Lower education was associated with lower likelihood of cancer suspicion: 2.6% of respondents with school-only education versus 7.3% with university education suspected cancer as a possible cause. In multivariable analysis, low education was the only demographic variable independently associated with lower cancer suspicion (odds ratio (OR) = 0.34, confidence interval (CI): 0.20–0.59).

**Conclusion:**

Levels of cancer suspicion were low overall in this community sample, and even lower in people from less educated backgrounds. This may hinder early symptomatic presentation and contribute to inequalities in stage at diagnosis.

## 1 Introduction

Studies in which cancer patients report retrospectively on the process of symptom appraisal indicate that not recognising a symptom as possibly due to cancer is an important determinant of delay in presentation [1–3]. Prolonged intervals from symptom onset to help-seeking may increase the risk of late stage diagnosis [4]. In Denmark and the United Kingdom (UK), where cancer survival rates are lower than other western countries with similar healthcare systems [5], there are ongoing campaigns to encourage public awareness of cancer ‘warning signs’ and prompt help-seeking [6,7].

People from lower socioeconomic status (SES) backgrounds are more likely to be diagnosed with later-stage disease for several cancer sites [8]. A number of factors potentially contribute to inequalities in stage of cancer diagnosis, but one that has attracted interest in recent years is how quickly people with symptoms present to their doctor (the so-called ‘patient interval’) [9]. Factors such as life stress and competing priorities – which tend to be higher in lower SES groups – have been considered as potential deterrents to prompt help-seeking [10], although as the overall primary care consultation rate is higher in lower SES groups, this is not a strong candidate for explaining long patient intervals [11]. An extended patient interval could also be due to individuals with lower levels of education being less equipped with the necessary ‘cancer literacy’ to recognise a cancer warning sign [10].

Surveys of public awareness of cancer, show that lower SES groups recall fewer cancer warning signs when tested with standardised psychometric measures [12–16]. However, this is ‘knowledge in theory’ and may not translate into differential symptom recognition in daily life. Evidence to date indicates that when people experience a warning sign in everyday life, very few suspect cancer [17], but there have been no studies examining SES differences in cancer suspicion in response to such a symptom.

In the present study, we combined data from two primary-care-based symptom surveys that used common methods of recruitment, and the same symptom assessments, to test the hypothesis that people with less education are less likely to suspect cancer when they experience a cancer ‘warning sign’.

## 2 Methods

### 2.1 Study population

Questionnaires were mailed to a total of 9771 men and women aged ⩾50 years, registered at seven General Practices across London, the South East and the North West of England, in surveys conducted in April 2012 and October 2013. Index of Multiple Deprivation 2007 (IMD 2007) scores at practice level were used to ensure a range of deprivation in participating practices. All patients registered at the participating practices who were ⩾50 years old, without a registered cancer diagnosis, and deemed suitable to complete the questionnaire by the doctor (e.g. did not have a mental illness, learning disability or terminal illness), were eligible. Non-responders were sent a reminder after 2 weeks. The study materials and protocol were approved by NHS London Bridge Research Ethics Committee (Reference: 11/LO/1970) and all patients gave informed consent.

### 2.2 Measures

#### 2.2.1 Demographics

The two surveys used the same questions on marital status (categorised for analysis as married/cohabiting versus not married/cohabiting), current employment (working versus not working), ethnicity (white versus non-white ethnic background) and education (university versus below university). Practices gave information on age and sex for each individual. Education was used as the marker of individual-level SES as it is considered more appropriate in an older sample, many of who are no longer in the workforce [18].

#### 2.2.2 Symptom experience and cancer attributions

Details of the questionnaire used in the first survey have been published [17]. Both questionnaires included questions on symptom experience phrased as: “*In the last 3 months have you had the following*” followed by a list of symptoms. The symptom list included the 10 symptoms from the Cancer Awareness Measure (CAM), which had been based on warning signs from Cancer Research UK’s website [19,20]. All had yes/no response options (see Table 2 for a full list of symptoms).

For each symptom that respondents had experienced, they were asked “*What do you think caused it?*” in a free-text response (termed open attribution item). ‘Cancer suspicion’ was defined as any instance where the respondent indicated that they had considered ‘cancer’ as a possible cause. People could give more than one attribution per symptom, and we coded any mention of cancer.

### 2.3 Data analysis

Descriptive statistics were completed for demographic characteristics, symptom frequency and symptom attributions. Non-responder analyses used chi-square and *t*-tests. Responses to the open attribution item were coded by two independent coders (KW and KeW), and divided into attribution categories [21]: ‘physical’, largely medical but excluding cancer (e.g. haemorrhoids for unexplained bleeding), ‘external/normalising’ (e.g. age for change in bladder habits), ‘psychological’ (e.g. stress for change in bowel habits), or ‘cancer.’ ‘Don’t know’ responses were counted separately, and blank responses were treated as missing. Cohen’s Kappa was used to assess the degree of agreement in rating symptom attributions, with coefficients >0.80 considered to represent good agreement [22]. Inter-rater reliability was high, ranging from Kappa = 0.80 (95% confidence interval (CI), 0.74–0.86) for persistent unexplained pain to Kappa = 0.93 (95% CI, 0.89–0.97) for change in the appearance of a mole.

Complex samples logistic regression analysis was used to investigate associations between socio-demographic characteristics and the likelihood of suspecting cancer. All 71 cancer attributions were included in the regression model using a ‘sampling with replacement’ (WR) design, and each participant’s identification number included as a random effect variable. Analyses were run with and without controlling for practice as a fixed categorical factor, but as there were no significant differences between the models, we report them without including practice. Data were analysed using the Statistical Package for the Social Sciences (SPSS) 22.0 [23].

## 3 Results

### 3.1 Participants

From 9771 people invited to take part in the survey, 3766 (38.5%) sent back a questionnaire, and 6005 (61.5%) did not reply after one reminder. Ten people did not complete the symptom questions and were therefore excluded from the analyses; resulting in a final sample for analysis of 3756. Demographic characteristics are presented in Table 1. Non-responder analysis showed that the probability of not responding was greater for men (37.0%) than women (40.3%) [*χ*^2^(1) = 10.90, *p* < .01], and for 50–59 year olds (33.1%) than 60–69 year olds (44.5%) or those 70 and over (40.4%) [*χ*^2^(2) = 100.25, *p* < .001].

### 3.2 Symptom experience

Nearly half the respondents (1732: 46.1%) had experienced at least one symptom from the cancer warning sign list in the past 3 months. The median number of symptoms reported was 1, the interquartile range was 1, and the full range was 0–10 (frequencies by warning sign are shown in Table 2). Persistent cough (16.9%) was the most common, and unexplained bleeding (2.9%) the least common.

Only a very small proportion (3.6%; 63/1732) of those who had experienced a ‘warning sign’ mentioned cancer as a possible cause. Six people suspected cancer for two of their symptoms, and one person made three cancer attributions, resulting in a total of 71 cancer suspicions. The distribution of cancer suspicion across symptoms is shown in Table 2. The highest number of cancer suspicions was for change in the appearance of a mole (10.7%). The lowest number was for change in bladder habits (0.7%).

### 3.3 Socio-demographic differences in cancer suspicion

To analyse socio-demographic differences in cancer suspicion, we examined the odds of suspecting cancer for any warning sign. In univariate analyses, lack of university education (odds ratio (OR) = 0.33, 0.19–0.55) was associated with being less likely to suspect cancer (see Table 3). There were no significant associations with sex, age, marital status, employment or ethnicity. Multivariate logistic regression analyses confirmed an independent effect of education (OR: 0.34, 0.20–0.59) after controlling for other demographic variables. See Table 3.

## 4 Discussion

This is the first study to examine socioeconomic differences in the likelihood of interpreting a ‘warning sign’ as suggestive of cancer. We used data from two waves of a ‘health’ survey to explore attributions in a large symptomatic sample. Despite the presence of public health campaigns in the UK, cancer suspicion was very unlikely in our respondents; only 3.6% mentioned cancer as a possible cause in the open text responses. However, as predicted, a lower level of education was associated with even lower likelihood of suspecting cancer. There were no differences by sex, age, employment, or marital status. There was a hint that respondents from non-white ethnic backgrounds were less likely to suspect cancer than those from white ethnic backgrounds, but it did not reach statistical significance. Predictably, ‘classic’ warning signs such as ‘unexplained lump’ or ‘change in the appearance of a mole’ were most likely to arouse cancer suspicion; however, even for those symptoms, the rates of cancer suspicion were little over 1 in 10. Persistent change in bladder habits and unexplained weight loss were the least likely to arouse cancer suspicion.

By combining data from two large health surveys, we had 1732 respondents reporting at least one ‘warning sign’ in the previous three months, but among these respondents the total number of cancer mentions was 71. We may therefore have lacked power to detect smaller socio-demographic effects. For the same reason, we had too few suspicions of cancer to explore socio-demographic effects at the individual symptom level. Previous work has suggested that SES differences in later-stage cancer diagnoses – which may be partly the result of late presentation – are concentrated in certain cancer sites [8]; suggesting that symptom-specific analyses would be useful. SES differences in late stage of diagnosis are greatest for cancers such as melanoma or breast, where patients typically present with easily noticeable symptoms (change in mole, unexplained lump) [8]. Nonetheless, even for these generally well-recognised symptoms, there are ‘knowledge gaps’ by SES [12–16]; consistent with the present findings of differences in cancer suspicion. However, we cannot rule out ‘denial’ as another explanation for the differences, and some people may have suspicions that they chose not to voice. A recent analysis of attitudes to a cancer diagnosis showed that lower SES respondents were more likely to believe that cancer treatment was worse than cancer itself, and less likely to want to know if they had cancer [24], which might underpin a reluctance to acknowledge cancer as a possible cause.

The participants in this study were drawn from a UK community sample population, but as the focus was on individual characteristics (e.g. symptom interpretation), our findings should be generalizable to other healthcare settings. Our study is relevant to other countries with similar General Practice systems, for example, those included within the International Benchmarking Partnership [25]. Response rates are almost always a limitation in community surveys. Our response rate was 39% which meant we had no information on the majority of potential respondents; although this is slightly higher than for some other community surveys [11]. In combination with the finding that men and younger people were less likely to respond, and previous evidence that non-responders are more likely to come from deprived residential areas [17], this limits generalisability. We also cannot estimate any response bias associated with symptom experience or interpretation; heightened attention to symptoms could either encourage or discourage questionnaire completion.

There was a significant proportion of missing data in the free-text attributions. This was a drawback, but at the same time it allowed us to identify people’s spontaneous attributions rather than prompting them with pre-defined categories; in keeping with our aim of investigating the symptom appraisal process without imposing the researcher’s cancer perspective. Another limitation was the use of simplistic categorisations for education and ethnicity. Future work should explore possible inequalities in likelihood of suspecting cancer in more heterogeneous samples.

The inherent challenge in the field of early diagnosis comes from the fact that most people experiencing ‘warning signs’ don’t have cancer. The low level of cancer suspicion by patients in this sample (3.6%) is similar to the average positive predictive value of cancer ‘warning signs’ [26,27], and similar to the levels of cancer suspicion observed by doctors themselves in primary care [28,29]. Despite this, encouraging more people to visit their doctors is an ongoing priority in the UK, supported both through public health campaigns [6], and general advice provided to the public by the National Health Service [30]. The logic is that these initiatives should result in more early stage diagnosis, and there is already some evidence for down-staging in the case of lung cancer [31], although conversion rates from secondary care referrals inevitably go down [32]. This means that more time is spent investigating people who don’t have cancer, and more people will experience a ‘false alarm’ which could undermine their likelihood of seeking help for a similar symptom in the future [33]. Our finding that people in general have low cancer suspicion when they experience ‘warning signs’, and that this is even lower in those more likely to be diagnosed at a later stage is important. People may need to be encouraged to lower their cancer suspicion ‘threshold’ through earlier diagnosis interventions, both at the public health and GP level.

One issue for consideration is the tension between encouraging people to think seriously about symptoms that could give an early warning of cancer and creating fear or hypochondriasis. One possibility is that people may not need to consider cancer as a cause *per se*. For example, recent research with colorectal cancer patients found that men who associated their symptoms with a potential illness (not necessarily cancer) were quicker to seek help than those attributing their symptoms to benign or self-limiting conditions [34]. Encouraging the public to seek medical advice for bodily changes that persist could therefore be a valuable approach.

However, it is known that cancer suspicion is a driver of help-seeking and our finding of inequalities in the likelihood of suspecting cancer when a ‘warning sign’ was experienced shows that a better understanding of the symptom recognition process could help to reduce inequalities in cancer survival.

## Role of funding source

The funders had no role in the study design, collection, analysis and interpretation of data, or of writing the report or in the decision to submit the article for publication.

## Financial support

These analyses were carried out with funding from the Department of Health Policy Research Unit in Cancer Awareness, Screening and Early Diagnosis awarded to J. Wardle and a Cancer Research UK Post-doctoral fellowship grant awarded to K.L. Whitaker (C33872/A13216).

The Policy Research Unit in Cancer Awareness, Screening and Early Diagnosis receives funding for a research programme from the Department of Health Policy Research Programme. It is a collaboration between researchers from seven institutions (Queen Mary University of London, University College London, King’s College London, London School of Hygiene and Tropical Medicine, Hull York Medical School, Durham University and Peninsula Medical School). The views expressed are those of the authors and not necessarily those of the NHS, or the Department of Health.

## Conflict of interest statement

None declared.

## Figures and Tables

**Table 1:** Demographic characteristics (% (*n*)).

		Total samplea,b *n* = 3756	Survey 1 *n* = 1723	Survey 2 *n* = 2033
Sex	Men	46.3 (1723)	46.2 (789)	46.5 (934)
	Women	53.7 (1996)	53.8 (920)	53.5 (1076)
Age (years)	50–59	34.6 (1273)	35.7 (609)	33.7 (664)
	60–69	37.3 (1374)	36.5 (622)	37.1 (752)
	70	28.0 (1030)	27.8 (474)	28.2 (556)
Education⁎	University	38.7 (1422)	40.8 (686)	36.9 (736)
	Below university	61.3 (2250)	59.2 (994)	63.1 (1256)
Employment⁎⁎	Working	42.7 (1587)	45.0 (769)	40.7 (818)
	Not working	57.3 (2129)	55.0 (939)	59.3 (1190)
Ethnicity⁎⁎⁎	White	88.5 (3293)	81.2 (1381)	94.7 (1912)
	Non-white	11.5 (428)	18.8 (320)	5.3 (108)

a Survey 1 respondents (London) had higher levels of university education, were more likely to be employed, and more likely to be from non-white ethnic backgrounds than Survey 2 respondents (London, South East and North West of England).

b Totals may vary due to missing data.

⁎ *p* < .05.

⁎⁎ *p* < .01.

⁎⁎⁎ *p* < .001.

**Table 2:** Experience of cancer ‘warning signs’ and symptom attributions.

	aPersistent cough or hoarseness	Persistent change in bowel habits	Persistent unexplained pain	Persistent change in bladder habits	Change in the appearance of a mole	Unexplained lump	Sore that does not heal	Unexplained weight loss	Persistent difficulty swallowing	Unexplained bleeding
% (*n*) reporting symptom	16.9 (629)	12.9 (483)	12.8 (476)	11.1 (413)	7.3 (273)	5.5 (205)	4.0 (148)	3.8 (143)	3.2 (120)	2.9 (108)
Attribution⁎ % (*n*)	(*n* = 545)	(*n* = 363)	(*n* = 370)	(*n* = 294)	(*n* = 178)	(*n* = 147)	(*n* = 114)	(*n* = 107)	(*n* = 87)	(*n* = 86)
Physical (non-cancer)	59.8 (326)	39.0 (142)	53.2 (197)	47.3 (139)	15.7 (28)	46.3 (68)	49.1 (56)	27.1 (29)	52.9 (46)	55.8 (48)
Psychological	3.1 (17)	8.5 (31)	4.3 (16)	3.7 (11)	0.6 (1)	2.0 (3)	1.0 (0.9)	18.7 (20)	9.2 (8)	3.5 (3)
External/normalising	33.8 (184)	45.7 (166)	27.8 (103)	41.2 (121)	44.4 (79)	17.0 (25)	28.1 (32)	44.9 (48)	16.1 (14)	20.9 (18)
Cancer	1.5 (8)	3.0 (11)	1.4 (5)	0.7 (2)	10.7 (19)	8.8 (13)	3.5 (4)	0.9 (1)	4.6 (4)	4.7 (4)
Don’t know	6.6 (36)	9.4 (34)	12.2 (45)	10.9 (32)	18.5 (33)	21.1 (31)	15.8 (18)	12.1 (13)	18.4 (16)	14.0 (12)

a Persistent was defined as ‘doesn’t go away’.

⁎ Each person could make more than one attribution and all attributions are included, so columns don’t add up to 100%.

**Table 3:** Prevalence, and unadjusted and adjusted odds ratios of suspecting cancer for one or more ‘warning sign’ in the past 3 months.

		Cancer suspicion *N* (%)	OR for suspecting cancer for any ‘warning sign’ (unadjusted), 95% CI	OR for suspecting cancer for any ‘warning sign’ (adjusted)⁎, 95% CI
Education	University (*n* = 563)	41 (7.3)		
	Below university (*n* = 841)	22 (2.6)	**0.33 (0.19–0.55)**	**0.34 (0.20–0.59)**
Sex	Men (*n* = 618)	27 (4.4)		
	Women (*n* = 803)	36 (4.5)	0.90 (0.54–1.48)	0.82 (0.50–1.36)
Age, years	50–59 (*n* = 474)	21 (4.2)		
	60–69 (*n* = 492)	24 (4.7)	1.19 (0.67–2.12)	1.15 (0.63–2.12)
	70+ (*n* = 401)	18 (4.6)	0.96 (0.51–1.82)	0.98 (0.47–2.05)
Employment	Working (*n* = 552)	27 (4.9)		
	Not working (*n* = 864)	34 (3.9)	0.87 (0.53–1.44)	1.11 (0.61–2.02)
Ethnicity	White (*n* = 1229)	61 (5.0)		
	Other (*n* = 188)	2 (1.1)	0.38 (0.11–1.29)	0.40 (0.11–1.42)
Marital status	Married/cohabiting (*n* = 812)	41 (5.0)		
	Not married/cohabiting (*n* = 607)	22 (3.6)	0.67 (0.40–1.12)	0.72 (0.42–1.24)

⁎ Adjusted for all other demographic variables reported in the table. Highlighted figures are statistically significant. OR = odds ratio, CI = confidence interval.
